# Pericyte infection by HIV-1: a fatal attraction

**DOI:** 10.1186/s12977-022-00614-3

**Published:** 2022-12-07

**Authors:** Oandy Naranjo, Silvia Torices, Paul R. Clifford, Manav T. Daftari, Olivia M. Osborne, Nikolai Fattakhov, Michal Toborek

**Affiliations:** Department of Biochemistry and Molecular Biology, University of Miami Miller School of Medicine, 528E Gautier Bldg. 1011 NW 15th Street, Miami, FL 11336 USA

**Keywords:** Pericytes, HIV-1 comorbidities, HIV, Brain

## Abstract

While HIV-1 is primarily an infection of CD4 + T cells, there is an emerging interest towards understanding how infection of other cell types can contribute to HIV-associated comorbidities. For HIV-1 to cross from the blood stream into tissues, the virus must come in direct contact with the vascular endothelium, including pericytes that envelope vascular endothelial cells. Pericytes are multifunctional cells that have been recognized for their essential role in angiogenesis, vessel maintenance, and blood flow rate. Most importantly, recent evidence has shown that pericytes can be a target of HIV-1 infection and support an active stage of the viral life cycle, with latency also suggested by in vitro data. Pericyte infection by HIV-1 has been confirmed in the postmortem human brains and in lungs from SIV-infected macaques. Moreover, pericyte dysfunction has been implicated in a variety of pathologies ranging from ischemic stroke to diabetes, which are common comorbidities among people with HIV-1. In this review, we discuss the role of pericytes during HIV-1 infection and their contribution to the progression of HIV-associated comorbidities.

## Introduction

One of the prevailing health concerns in HIV-1 management is that virally suppressed patients remain at increased risk of HIV-associated comorbidities. Indeed, several epidemiological studies have delineated a higher susceptibility to mental health, neurodegenerative, respiratory, and cardiovascular diseases in HIV-1 infected individuals [1–4]. Among comorbidities, ~ 56% people living with HIV (PLWH) develop hypertension, 2–15% develop chronic obstructive pulmonary disease, 15–20% suffer from depression, 1–5% experience a stroke in their lifetime, and 4–34% show ischemic lesions at autopsy [5–9]. Emerging evidence indicates that HIV-1 infection is not limited to T-cells or monocytes/macrophages, but it affects a variety of different cell types in several compartments of the body [10–14]. Indeed, it has become apparent that understanding HIV-1 infection in non-T cells is key to treating comorbidities that persist at low viral levels. Several non-T cell reservoirs have been discovered including astrocytes, microglia, dendritic cells, and pericytes [10–12, 15–20]. We propose that by studying the interactions of HIV-1 with pericytes from different vascular beds, we may better design and treat vascular comorbidities arising from this complex cellular interplay.

**Fig. 1:**
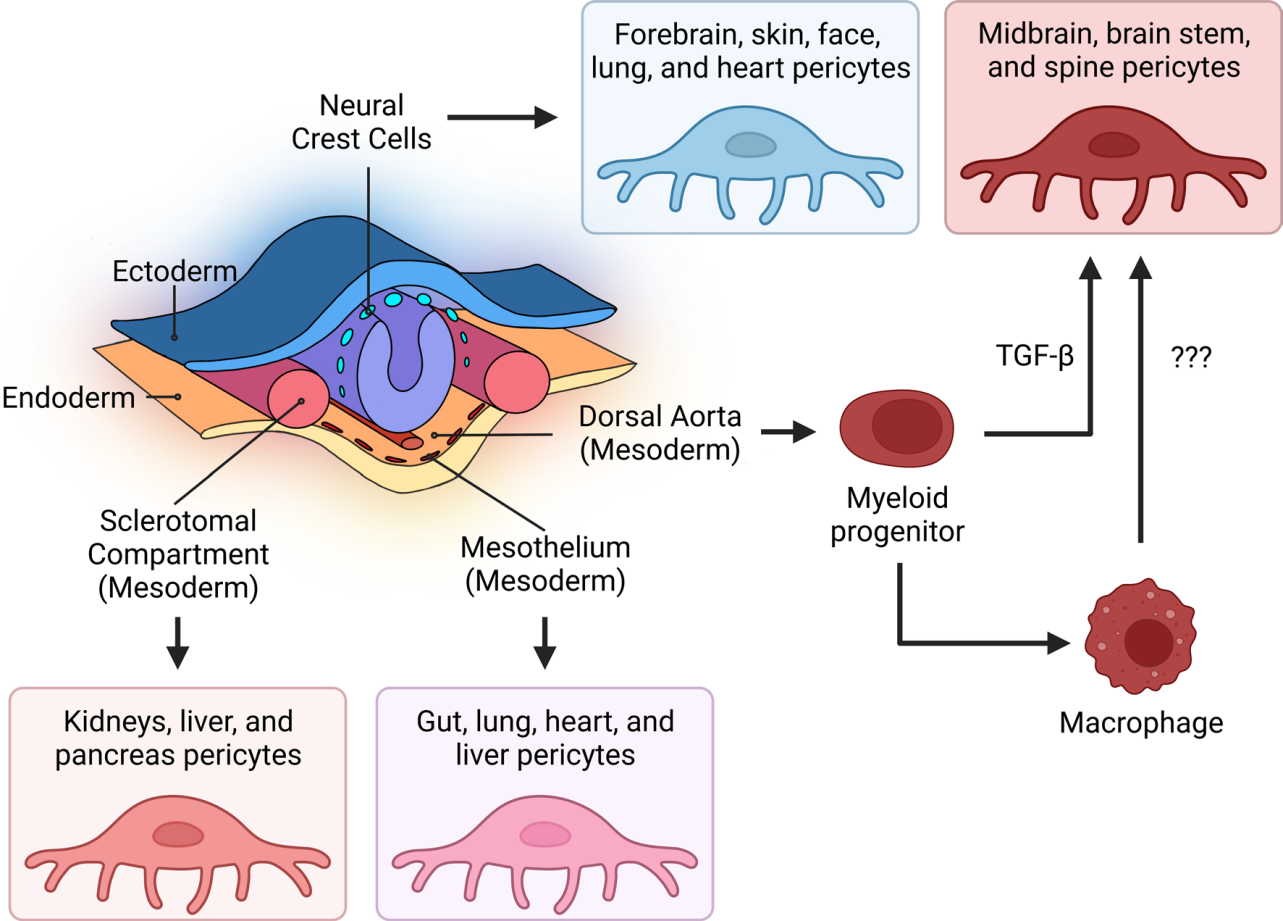
Heterogeneity of pericyte origins throughout the body

Pericytes are mural cells surrounded by a basement membrane, situated on the exterior of the endothelial cells in capillaries, pre-arterioles, collecting venules, and pre-venules [21]. Despite their physiological importance, a complete understanding of pericyte developmental origins remains elusive. Indeed, pericytes are heterogenous throughout the body and even within specific tissues. Ectodermal tissue gives rise to forebrain, skin, lung, face, and heart pericytes and mesoderm gives rise to all other tissues. Even within the population of mesodermal pericytes there are different subsets including the sclerotomal compartment, mesothelium, and dorsal aorta [22, 23]. Pericytes can express multiple markers and have a high degree of plasticity, which lead to difficulties when determining their ontogeny [24] **(**Fig. 1**)**. The importance of pericyte morphology and function vary depending upon their location in the vascular tree, and how they have differentiated. However, understanding their signaling and structural pathways may aid us in targeting clinical comorbidities that manifest during HIV-1 infection [25–27].

A typical function of pericytes is regulation of capillary blood flow, endothelial maintenance, and regulation of immune cell entry. To regulate capillary blood flow, thin processes of pericytes surround vessels and contract or expand, which results in a decrease or increase in the cross-sectional area of the vessel cavity, and modulation of flow rate [28]. A change in the cells’ membrane potential and a subsequent influx of calcium ions triggers an interaction between ɑ-smooth muscle actin filaments (ɑ-SMA) and myosin to create mechanical tension on the extracellular matrix of the pericyte, contracting the cell [29]. Due to the metabolic demands of contraction events, pericytes require high numbers of mitochondria and are highly susceptible to mitochondrial dysfunction. In addition to blood flow regulation, pericytes communicate with neighboring endothelial cells that form the basement membrane of the blood vasculature [25]. The pericyte-endothelial interface is composed of several important signaling pathways responsible for mutual cell survival. Pericytes secrete angiotensin which promotes barrier stability and in turn, endothelial cells secrete platelet derived growth factor-B (PDGF-B) which promotes pericyte stability and survival. The anti- and pro-apoptotic protein, B-cell lymphoma-w (BCL-w), is secreted by pericytes and stimulates vascular endothelial growth factor A (VEGF-A) expression in endothelial cells and can ultimately halt unnecessary apoptosis [30]. Additionally, the process of angiogenesis is, in part, mediated by the interplay between Neuron-glial antigen 2 (NG2) and β1 integrin transmembrane receptors, and the formation of gap junction proteins maintaining vessel integrity [27, 31, 32].

**Fig. 2:**
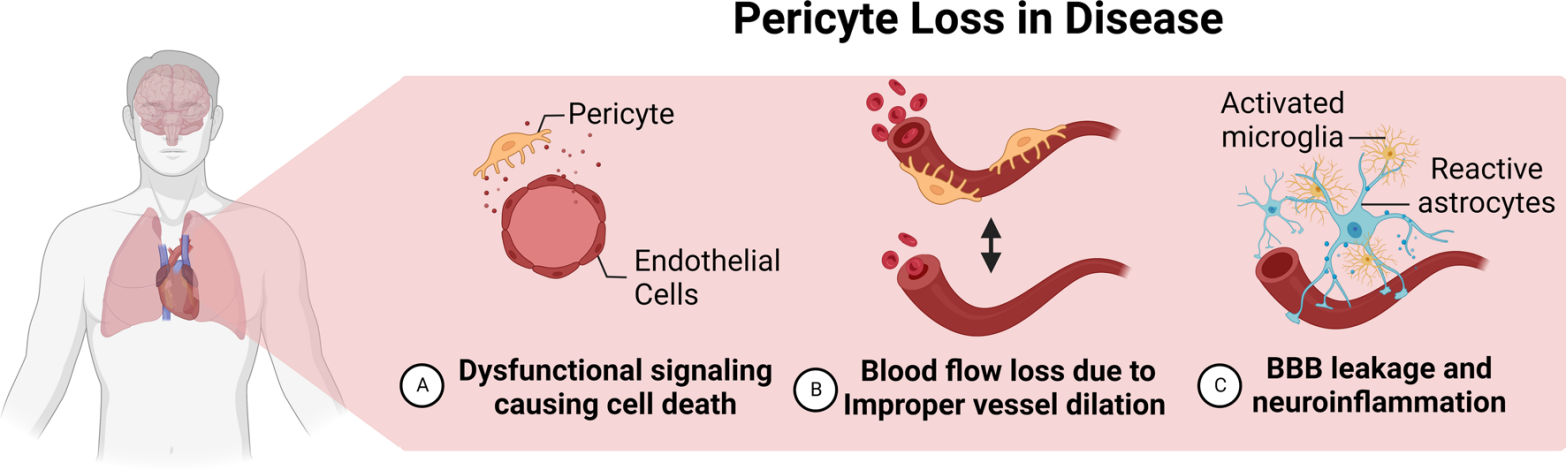
Physiological effects of pericyte loss in function and disease

Pericytes play a role in microvascular circulation notably in the lungs and striated muscles. However, the most prominent density of endothelial cell coverage by pericytes is in the brain, where they cover over 90% of the endothelium [33, 34]. Central nervous system (CNS) pericytes contribute to the structure of the neurovascular unit (NVU), a functional component of the blood-brain barrier (BBB) and the blood-retinal barrier (BRB). Pericytes play a key role in the immunological function of these systems. By reducing the expression of signaling proteins that increase vascular permeability, pericytes reinforce the structural integrity of the BBB and BRB; thereby, preventing neuroinflammation via endothelial cell-mediated transmigration of leukocytes. Loss of pericyte coverage and function represents a significant challenge for the body. A decrease in pericyte coverage has been observed in several diseases, including diabetic retinopathy, hypertension, kidney disease, and stroke [8, 35–40] **(**Fig. 2**)**.

The vascular system is critical to every organ in the body, disseminating nutrients and oxygen to maintain proper function and the pericyte-endothelial interface is actively remodeled during angiogenesis, development, and during acute and chronic vascular disorders [6, 41, 42]. Viral infection of pericytes can lead to vascular leakage, increased viral exposure due to barrier breakdown, including the CNS, as well as inflammatory, neurological, cognitive, and developmental effects. Indeed, several HIV-1 associated comorbidities are cardio- or cerebrovascular related or include a breakdown of the vascular system. Here we summarize the current evidence for pericytes as key contributors to HIV-1 infection and how pericyte dysfunction leads to HIV-associated complications.

## Pericytes and brain infection by HIV-1

Brain pericytes are part of a microvascular system that forms the BBB, a highly selective semipermeable interphase between the blood stream and brain parenchyma [25, 43]. Pericytes are highly heterogeneous, their origin varies in different parts of the body and even in various regions of the brain [22, 23]. While pericytes in the forebrain are derived from neural crest cells, pericytes in the rest of the brain appear to originate from mesenchymal stem cells of the mesoderm [44]. Moreover, recent evidence indicates that myeloid progenitors, arising from dorsal aorta mesoderm, also contribute to the development of pericytes in the brain. In fact, it was proposed that a substantial pool of brain pericytes originate from yolk-sac-derived macrophage progenitors [45] **(**Fig. 1**)**.

**Fig. 3:**
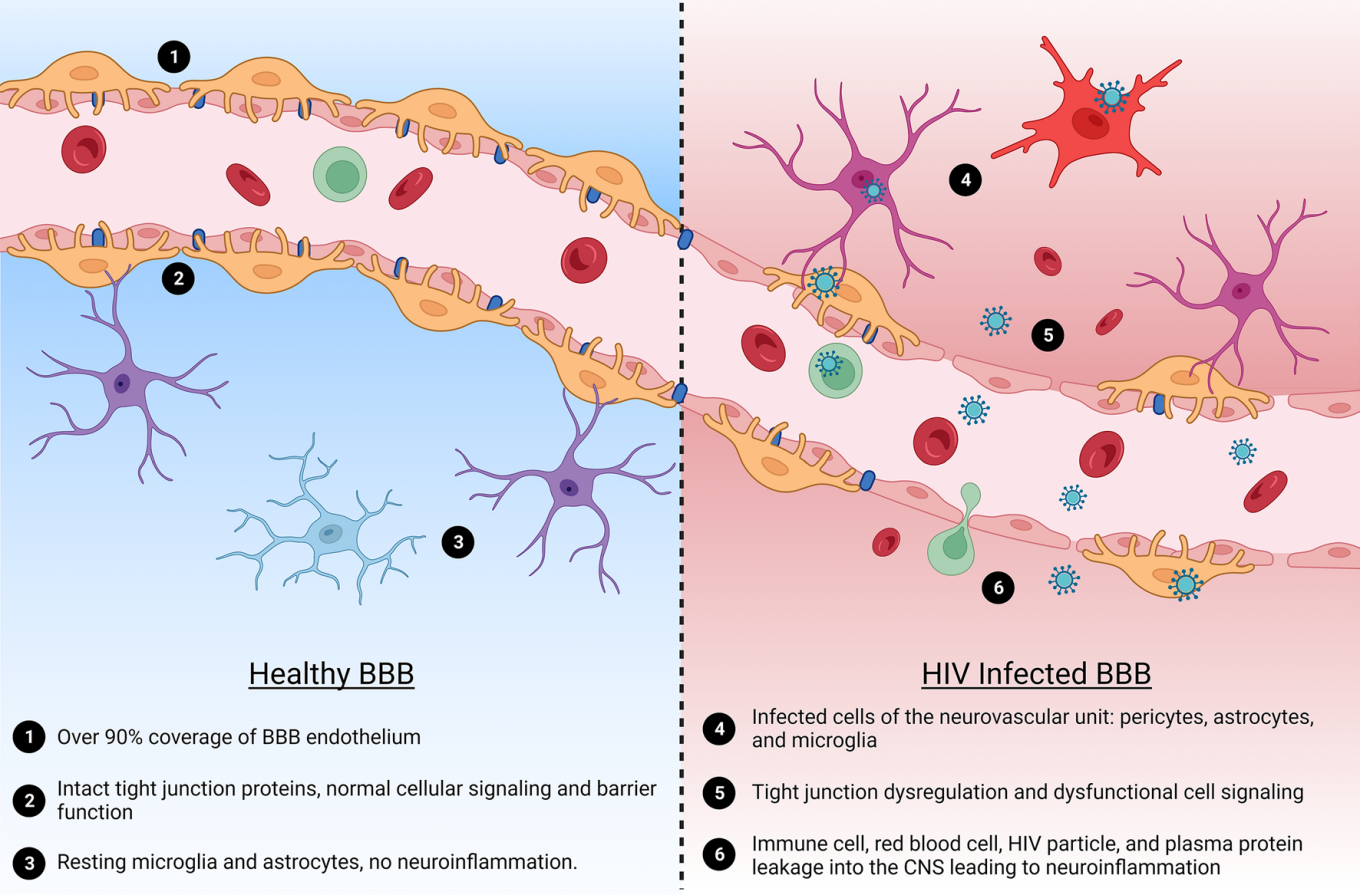
Understanding the effects of HIV-1 on blood brain barrier physiology

The neurovascular unit (NVU) of the BBB is composed of brain microvascular endothelial cells, pericytes, and astrocytes [46, 47]. Tight junction (TJ) proteins link endothelial cells together and form a selective permeable barrier between the blood and the CNS, which prevents toxic molecules, viruses, bacteria, and inflammatory cells from reaching the brain [34, 48]. Transporter proteins on both sides of BBB keep homeostasis by allowing for the selective exchange of nutrients into the brain and of toxic substances out. By using different pathways, HIV-1 can cross the BBB early after infection and enter the brain parenchyma, leading to a persistent infection of the CNS [20, 49–51]. The protected nature of the CNS is a major challenge for treating HIV-1 infection because of anti-retroviral therapy (ART) drugs’ inability to efficiently cross the BBB and accumulate in the brain parenchyma [52, 53]. Lower ART concentrations in the CNS allow the virus to accumulate in the brain resulting in a progressive cycle of cell damage and repair [52]. Over the lifetime of the individual, cell damage and subsequent neuroinflammation caused by HIV-1 infection can progress to HIV-associated neurocognitive disorders (HAND) [54, 55] **(**Fig. 3**)**.

While the role of endothelial cells in the formation of the BBB has been recognized, emerging evidence indicates a critical role for pericytes in the maintenance of BBB functions [56–58]. BBB pericytes have been shown to regulate paracellular and trans-endothelial fluid transport, maintain homeostasis of the microenvironment, and protect endothelial cells [59]. Additionally, BBB pericytes have been shown to play a key role during viral infections such as HIV-1 infection in the brain [60, 61].

The mechanisms of HIV-1 infection of pericytes remain elusive despite significant progress achieved in recent years. Within 48 h of pericyte infection by HIV-1, there is a significant increase in viral replication, which is associated with NFKB acetylation and a decrease in occludin expression. These events have been linked to activation of the SIRT-1 pathway, which is integral to controlling NFκB acetylation. HIV-1 hijacking of the SIRT-1 pathway for viral replication is well established in other cells; however, this process in pericytes appears to be accomplished by depleting cellular occludin levels. Indeed, overexpression of occludin leads to a decrease in viral loads and diminished SIRT-1 activation [41, 62]. Additionally, several experiments assessing HIV-1 replication show that BBB pericytes can be productively infected by HIV-1 in vivo and in vitro [12, 20, 60, 63]. Indeed, BBB pericytes express CD4, the main HIV-1 receptor, and prominent levels of the HIV-1 co-receptors, CCR5 and CXCR4, which allows them to be directly infected by both X4 and R5 tropic HIV-1 strains [12]. Measure of reverse transcriptase activity and p24 levels from supernatant of infected cells reveals that pericytes exhibit the highest viral replication 2–3 days post HIV-1 infection, followed by a steady decrease after 7–10 days post-infection. Furthermore, BBB pericytes exhibit a correlation between a decrease in HIV-1 production and an increase in HIV-1 host genome integration. Moreover, an increase in p24 and HIV-1 RNA production by latently infected pericytes can be achieved by exposure to histone deacetylase inhibitors and tumor necrosis factor [20]. Altogether, the evidence points to BBB pericytes as capable of active and latent infection and representing a potential target for an HIV-1 reservoir in the CNS.

Several studies have shown a correlation between HIV-1 brain infection, neurological damage, and an increase in BBB permeability [38, 50, 64, 65]. Alterations of BBB integrity after HIV-1 infection are associated with changes in TJ protein expression and an increase in proinflammatory responses. Specifically, HIV-1 infection of pericytes causes a decrease in the expression of TJ proteins, such as occludin and ZO-1, mitochondrial dysfunction, and IL-6 production [41, 66, 67]. During HIV-1 infection, there is also a significant decrease in BBB pericyte coverage of the brain endothelium [12, 20, 65, 68], which can potentiate alterations to BBB integrity that facilitates the HIV-1 penetration into the CNS [50, 69, 70]. Additionally, pericyte to endothelial cell-to-cell communication is critical for the maintenance of endothelial cells and TJ integrity. Pericyte cellular signaling has been shown to change during HIV-1 infection. Injury signals are propagated from infected pericytes to neighboring cells via gap junction (GJ)-mediated intercellular communication and occludin, caveolin-1, and alix, which form a multi-protein complex (cav-1-ocln-alix) that alters pericyte gene expression and membrane plasticity following infection [60, 63]. These molecular changes in BBB pericytes have direct outcomes on cerebrovascular health. BBB disruption caused by HIV-1 infection has been shown to potentiate stroke sizes and worsen post stroke recoveries in mouse models infected with EcoHIV [68]. EcoHIV is a chimeric virus that was generated by replacing the coding region of the surface envelope glycoprotein, gp120, in HIV-1 with the envelope-coding region (gp80) from ecotropic murine leukemia virus, a retrovirus that infects only rodents, enabling EcoHIV to use mice as a host and restricting EcoHIV from infecting human cells [71]. Moreover, HIV-1 infected individuals are at a higher risk for several cerebrovascular/neurological comorbidities and understanding HIV-1 infection of BBB pericytes may be an important step to improving their quality of life [42, 64, 68, 72].

## HIV-1 infection of Peripheral Pericytes

Pericytes’ coverage of the microvascular endothelium is not limited to the brain but extends to peripheral tissues, making them potentially vulnerable to HIV-1 infection and HIV-associated comorbidities. While a pool of CNS pericytes share an origin from neural crest, most peripheral pericytes are derived from mesoderm, splitting the body into two crude subsets of mural cells **(**Fig. 1**)**. Indeed, the presence of HIV-1 in pericytes defined by PDGFRβ expression has been confirmed in vitro in cultured human lung pericytes and in vivo in lung tissue of SIV-infected macaques. Lung pericytes of both macaques and humans express the necessary receptor profile for HIV-1 infection [73]. Virally unsuppressed macaques exposed to SIV infection show detectable levels of HIV p24 production colocalizing to PDGFRβ in isolated lung pericytes. Infected human lung pericytes cultured in vitro produce functional viral particles capable of infecting a T cell line exposed to conditioned media, demonstrating viral transmission [73]. Disruption of pericyte function may contribute to chronic lung pathologies seen in HIV-1 infected individuals, including chronic obstructive pulmonary disease which is diagnosed in up to 20% of patients [9, 74, 75].

Human retinal and brain tissue arise from the same embryonic origin, suggesting that the BRB may be similarly susceptible to HIV-induced alterations as the BBB [76]. While productive HIV-1 infection of retinal pericytes has yet to be confirmed, one of the most common reasons for vision loss in patients with HIV-1 is diabetic retinopathy [38, 77]. Retinopathy is a disease categorized by increased vascular permeability and progressive vascular occlusion to the vessels in the eye. One of the hallmarks of retinopathy is a loss of pericyte coverage at the BRB causing morphological changes and barrier dysfunction [35, 36]. Using evidence from the BBB we know that HIV-1 can cause a loss of BBB pericytes and a significant increase in BBB permeability. Understanding how HIV-1 and hyperglycemia affect BRB pericytes may be critical to design novel therapeutics for HIV-associated retinopathy.

Current reports of brain and lung infection suggest that both neural crest- and mesoderm-derived pericytes are susceptible to HIV-1 infection. The intersection of HIV-1 and pericyte research is a new field and with several HIV-associated comorbidities revolving around vascular damage (hypertension, stroke, heart disease) pericytes across the body may become important players in the treatment of HIV-1.

## Modulation of pericyte biology by HIV-1

**Fig. 4:**
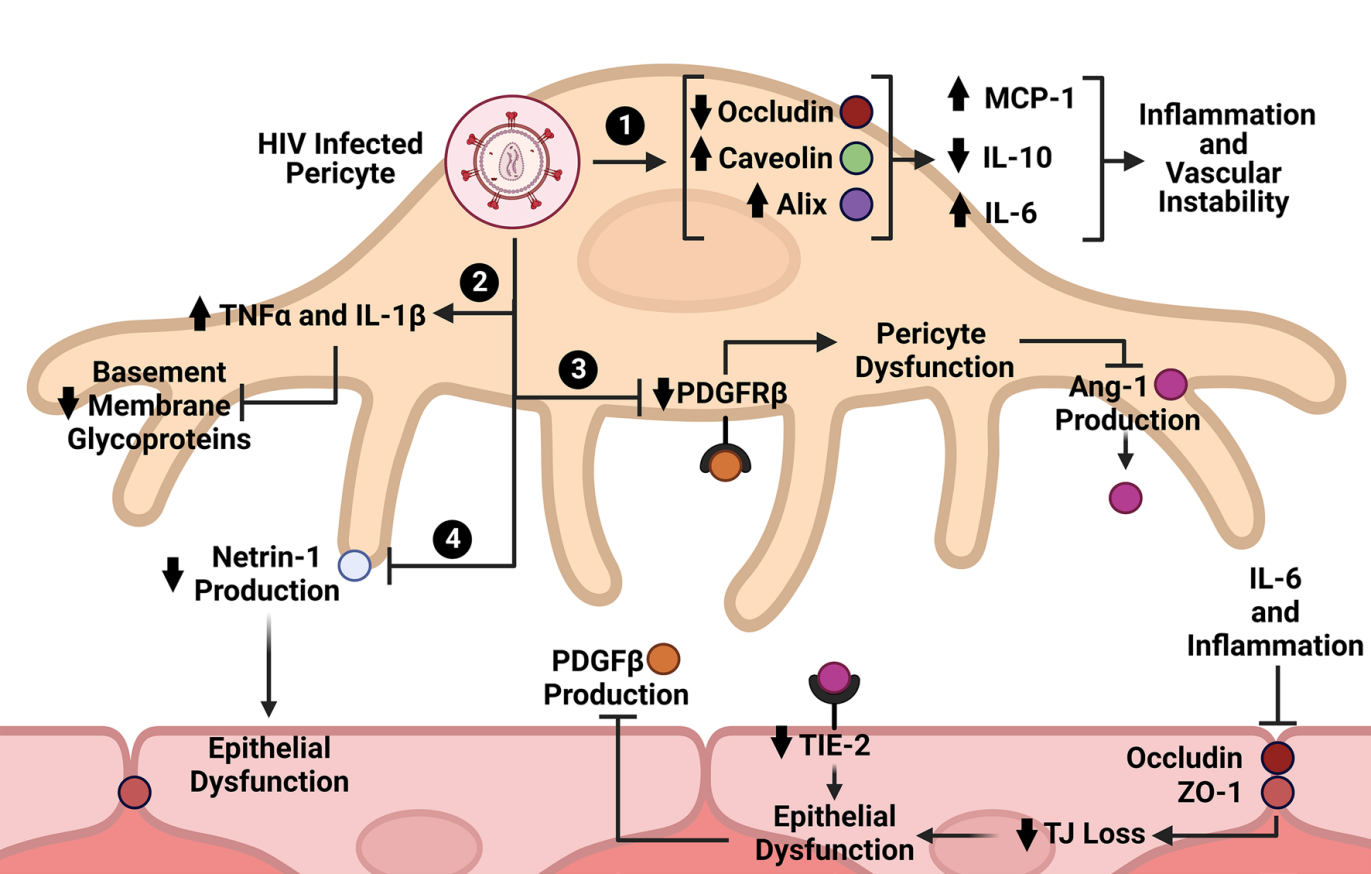
Summary of major effects of HIV-1 on pericyte signaling

Due to the high incidence of HIV-1 associated comorbidities and neurological disorders, defining how HIV-1 modulates cellular pathways in pericytes is of critical relevance to translational medicine. Several molecules in key pathways of cell survival, migration, and metabolism have shown to be significantly modulated during HIV-1 infection of BBB pericytes. Exposure of pericytes to HIV-1 significantly decreased occludin levels 48 h after infection, followed by significant increases in caveolin-1 and alix expression which steadily climbed to both 48 and 72 h after infection. Modulation of the cav-1-ocln-alix complex by HIV-1 can then contribute to decreased levels of IL-10, IL-15, INF-γ, and G-CSF and enhanced expression of IL-6, and MCP-1/CCL-2. Increases in IL-6 and decreases in IL-10 levels correspond to a pro-inflammatory profile consistent with studies of HIV-1 in other cell types [63]. Interestingly, IL-6 is a major driver of neuroinflammation, which decreases expression of TJ proteins occludin and ZO-1 in endothelial cells, leading to a decrease in BBB integrity [63, 78–80] **(**Fig. 4**).**

Similarly, when exposed to pro-inflammatory factors, pericytes show a 50–60% decrease in platelet derived growth factor receptor (PDGFRβ) expression. PDGFRβ downstream signaling is involved in pericyte survival and maintenance; in contrast, loss of PDGFRβ leads to a decrease in pericyte coverage of endothelial cells and a decrease in angiopoietin-1 (Ang-1) production. Ang-1 is responsible for Angiopoietin-1 receptor (TIE-2) activation in endothelial cells and is an important regulator of endothelial quiescence and activation. Loss of Ang-1 leads to BBB instability and decreased pericyte coverage. The PDGFRβ-TIE-2 axis is just one example of the delicate crosstalk that pericytes and endothelial cells share to maintain survival and how HIV-1 infection may lead to disruption of this balance [63, 81] **(**Fig. 4**).**

Additionally, pericytes exposed to HIV-1 increase the production of tumor necrosis factor alpha (TNF-α) and interleukin-1beta (IL-1β). TNF-α and IL-1β are relevant molecules to HIV-1 infection and have several downstream effects on both cellular metabolism and migration. Pericytes challenged by HIV-1, TNF-α, or IL-1B show a 60% downregulation in basement membrane glycoproteins fibronectin (FN), nidogen-1 (NID-1), nidogen-2 (NID-2), and show an increased migratory phenotype [81]. Pericytes and endothelial cells are responsible for basement membrane synthesis [25] and basement membrane abnormalities are known to be present in several neurodegenerative and neurovascular diseases [21, 82, 83] **(**Fig. 4**).** Lastly, increased extracellular glutamate caused by neuroinflammation acts as a neurotoxin leading to mitochondrial dysfunction, reactive oxygen species generation, oxidative DNA damage, and cell death [84, 85]. During ischemic stroke and diabetic nephropathy, both BBB and BRB pericytes show sensitivity to glutamate excess. Pericytes cultured with TNF-α exhibit increased susceptibility to glutamate leading to mitochondrial dysfunction and oxidative stress, potentiating neuroinflammation and pericyte loss at the BBB [20, 86–88]. Modulation of key pathways by HIV-1 has several implications for the blood vasculature and their surrounding tissues in the CNS and other organ systems.

## Discussion, limitations, and conclusions

Among HIV-1 associated comorbidities, cardiovascular and cerebrovascular pathologies are prevalent, highlighting the importance of vascular biology for the long-term outcome of the infection. For HIV-1 to enter tissues, virions must cross the blood vasculature via direct or indirect interactions with endothelial cells and/or pericytes. Recent work has shown that pericytes possess the necessary receptor profile for HIV-1 infection. Productive HIV-1 infection in pericytes has been confirmed using post-mortem brain samples, in vivo SIV and EcoHIV infection, and in vitro work, while latent infection of pericytes has only been suggested based on in-vitro observations. CNS pericytes derive from neural crest while lung pericytes have mesodermal origins, together the neural crest and mesoderm encompass the origin of all pericytes in the body. Therefore, HIV-1 infection of both groups of pericytes reported in the literature suggests that all pericytes, independent of origin, are susceptible to HIV-1 infection. Additionally, HIV-1 infection is capable of significantly modulating pericyte survival, metabolism, and migration pathways. Infected pericytes adopt a pro-inflammatory state that has ramifications for cells that depend on pericyte cellular signaling. Conditioned media from HIV-1-infected pericyte culture has been shown to disrupt endothelial cell survival and have a potential to activate microglia and astrocytes, leading to an inflammatory cascade.

There are remaining important limitations to the current studies of HIV-1 and pericyte infection. To date, all post-mortem tissues that have been analyzed for HIV-1 infection of pericytes have been obtained from infected individuals without ART treatment. Thus, there are no published results evaluating HIV-1 pericyte infection in post-mortem brain samples from virally suppressed patients. Nonetheless, the current studies provide proof of principle that pericytes of different origin and from different compartments are capable of infection and are a noteworthy cell type in the study of HIV-1. Pericytes are ubiquitous to every organ system and understanding the mechanisms of their infection may carry the potential to fill in several important gaps necessary to improve the quality of life for people living with HIV-1.

## Data Availability

Available upon request.

## Abbreviations

Ang-1: Angiopoietin-1
ART: Anti-retroviral therapy
BBB: Blood-brain barrier
BCL-w: B-cell lymphoma-w
BRB: Blood-retinal barrier
CNS: Central nervous system
FN: Fibronectin
GJ: Gap junction
HAND: HIV-associated neurocognitive disorders
IL-1β: Interleukin-1β
NG2: Neuron-glial antigen 2
NID: Nidogen
NVU: Neurovascular unit
PDGF-B: Platelet derived growth factor beta
PLWH: People living with HIV
PDGFRβ: Platelet derived growth factor receptor beta
ɑ-SMA: ɑ-smooth muscle actin
TJ: Tight junction
TNF-α: Tumor necrosis factor-α
VEGF-A: Vascular endothelial growth factor A
